# Sugar-Free Dark Chocolate Consumption Results in Lower Blood Glucose in Adults With Diabetes

**DOI:** 10.1177/11786388221076962

**Published:** 2022-02-07

**Authors:** Barbara Oliveira, Kaja Falkenhain, Jonathan P Little

**Affiliations:** School of Health and Exercise Sciences, University of British Columbia, Kelowna, Canada

**Keywords:** Diabetes, chocolate, glucose

## Abstract

Diabetes is characterized by an impaired ability to appropriately control blood glucose. Postprandial hyperglycemia, in particular, is associated with complications in people with type 1 diabetes (T1D) and type 2 diabetes (T2D). The objective of this study was to determine how sugar-free dark chocolate sweetened with stevia, erythritol, and inulin impacts postprandial blood glucose levels in individuals with diabetes compared to conventional dark chocolate. In a randomized crossover design, 13 participants consumed 1 bar (34 g) of sugar-free dark chocolate or 1 bar (34 g) of conventional dark chocolate with glucose levels measured before and throughout a 120-min postprandial period. The incremental area under the curve (iAUC) was lower after the consumption of sugar-free dark chocolate (−65%, *P* = .04) compared to conventional dark chocolate. No significant differences between chocolates were found for peak glucose value above baseline, the total area under the curve, or peak glucose values. Our results suggest that a sugar-free dark chocolate bar sweetened with stevia, erythritol and inulin led to a lower blood glucose iAUC compared to the conventional dark chocolate bar in people with diabetes, whilst longer-term effects on glucose control remain to be determined.

## Introduction

Diabetes is a growing concern around the world that affects an estimated 9.3% of adults, ages 20 to 79, worldwide.^1^ The situation in Canada is similar, with a prevalence of ~3.4 million people (9.3% of the population).^2^ Approximately 10% of diabetes cases are type 1 diabetes (T1D) while the majority (~90%) are type 2 diabetes (T2D). Both major types of diabetes are characterized by a impaired ability to control blood glucose and are diagnosed with the same hyperglycemic thresholds. Diet is clearly an important consideration for people with diabetes, and a main recommendation for people with both types of diabetes is to limit sugars and sweets, as they may cause a high postprandial blood glucose response.^3^

In diabetes, the postprandial phase is characterized by a rapid and large increase in blood glucose levels, and accumulating evidence indicates that these postprandial “hyperglycemic spikes” contribute to the pathophysiology of diabetes complications, particular vascular dysfunction.^4^ Therefore, limiting postprandial hyperglycemia is an important therapeutic strategy to minimize microvascular and macrovascular complications of diabetes.^5^

Adults with diabetes often avoid chocolate due to its sugar content; however, high polyphenol dark chocolate has been shown to mitigate the deleterious effects of hyperglycemia on blood vessel function in people with diabetes.^6^ Furthermore, not all types of chocolate lead to the same glucose response; In a study that examined the consumption of isocaloric doses of dark, milk, and white chocolate, blood glucose levels were higher 30 min after ingestion of white and milk chocolate compared to the dark chocolate.^7^ This suggests that people with diabetes may consider selecting dark chocolate products to minimize the impact on glucose control. Daily consumption of flavonoid-rich dark chocolate was shown to improve fasting plasma glucose levels and insulin resistance to an extent three times greater than milk chocolate.^8^ The 15-day administration of polyphenol-rich dark chocolate was also shown to reduce blood pressure and increase insulin sensitivity in healthy participants.^9^ Similar results were observed after 4 weeks of consuming dark chocolate in participants with overweight and obesity.^10^ The health benefits of chocolate appear to be primarily driven by the polyphenol and cocoa content, which are greatest in dark chocolate.

The use of sweeteners instead of sugar in dark chocolate could be an attractive option for people with diabetes as such a formulation would not be expected to spike blood glucose. A study evaluating the consumption of sucrose, fructose and isomalt-sweetened chocolates in participants with type 2 diabetes found that while sucrose and fructose-sweetened chocolates induced a similar glycemic effect as sugar, isomalt-sweetened chocolate produced a lower glucose excursion.^11^

Determining the blood glucose response to sugar-free dark chocolate could provide evidence that this type of chocolate is an acceptable food choice for people with diabetes that could promote the potential vascular benefits of consuming dark chocolate without negatively impacting blood glucose control.

The objective of this study was to determine how sugar-free dark chocolate sweetened with stevia, erythritol, and inulin impacts blood glucose levels in individuals with diabetes as compared to a conventional dark chocolate sweetened with sugar. It was hypothesized that the sugar-free dark chocolate bar would lead to a lower postprandial blood glucose response compared to the conventional dark chocolate bar.

## Materials and Methods

### Study design

A randomized double-blind crossover pilot trial was performed. The study was approved by the Clinical Research Ethics Board of the University of British Columbia (UBC CREB number H20-02122, date of approval 24 November 2020). The study was registered on ClinicalTrials.gov (Identifier: NCT04847999) on 19 April 2021. Due to the COVID-19 curtailment of in-person research at our institution, the study was conducted remotely, since trial participants were unable to come to the investigational site for protocol-specified visits. Information and guidance for the trial were delivered by email, telephone calls, and video meetings. RedCap-UBC, a secure web application to build and manage online surveys and databases, was used to manage information and provide questionnaires. Written informed consent for inclusion was obtained digitally from all subjects before enrollment.

Participants received a box containing a OneTouch Verio IQ® blood glucose monitoring system (OneTouch Verio IQ^®^ meter, lancing device, sterile lancets, carrying case), glucose strips, alcohol swabs, 1 bar of sugar-free dark chocolate (34 g), 1 bar of a conventional dark chocolate (34 g), and instructions on how to proceed during each trial. To achieve double-blinding, chocolate bars were prepared in the same mold, packaged in identical wrappers, and labeled A and B by Ross Chocolates Inc. (Port Coquitlam, BC, Canada). The identity of the chocolate bars was not revealed to the research team until after all data analyses. Participants completed 2 experimental trials: (1) consumption of 1 bar (34 g) of sugar-free dark chocolate and (2) consumption of 1 bar (34 g) of conventional dark chocolate. The order of the bars was randomized for an equal chance of getting bar A or bar B first and trials were performed 3 to 7 days apart. Participants were advised to do both trials on the exact same time of day, in the morning after an overnight fast, and to refrain from taking diabetes medications the morning of the trial. They were asked to register glucose levels before and after eating the chocolates. The OneTouch Verio IQ^®^ meter was used to measure blood glucose. The time points were as follows: baseline (immediately before chocolate consumption) and 15, 30, 45, 60, 90, and 120 min after chocolate consumption. Values were registered by the participant on a prepopulated data form that was sent by the research team using a RedCap-linked email and were also stored on the meter for verification.

### Participants

Thirteen (N = 13) individuals with physician-diagnosed T1D (N = 6) and T2D (N = 7) were recruited through online social media and newspaper advertising. Inclusion criteria were: (i) physician-diagnosed T1D or T2D of ⩾1 year; (ii) current HbA1c of 6.5% to 8.5%; (iii) BMI: 25 to 40 kg/m^2^; (iv) blood pressure of <160/99 mm Hg; (v) non-smoking; (vi) not on hormone replacement therapy, corticosteroids, or anti-inflammatory medications; and (vii) 18 to 75 years old. Exclusion criteria included: (i) taking more than 2 glucose lowering medications; (ii) ongoing medical treatment for diseases such as cancer, autoimmune or inflammatory diseases, liver or kidney disorders; (iii) allergy, intolerance or aversion to cocoa, stevia, erythritol, inulin, or any other dietary restrictions (eg, vegan) that prevented them from consuming standardized study foods; (iv) unable to follow remote guidance via the Internet or smartphone; (v) unable to follow controlled diet instructions; (vi) unable to read or communicate in English.

### Chocolate bars

According to the manufacturer, 34 g of sugar-free dark chocolate provided 2 g of protein, 15 g of fat and 16 g of carbohydrates (8 g of fiber and 5 g of sugar alcohols), thus delivering 150 kcal. Conventional dark chocolate was chosen to be as closely matched as possible and provided 3 g of protein, 14 g of fat and 16 g of carbohydrates (5 g of fiber and 10 g of sugar), thus providing 193 kcal. Detailed nutritional information is presented in Table 1.

**Table 1.** Nutritional composition of sugar-free dark chocolate and conventional dark chocolate.

	Sugar-free dark chocolate	Conventional dark chocolate
Grams (g)	34	34
Calories (kcal)	150	193
Fat (g)	15	14
Saturated fat (g)	9	8
Trans fat (g)	0	0
Cholesterol (mg)	0	0
Sodium (mg)	0	11
Carbohydrates (g)	16	16
Fibers (g)	8	5
Sugars (g)	0	10
Protein (g)	2	3
Cocoa (%)	66	70

Information obtained from the manufacturer and the packaging of each chocolate.

The sugar-free dark chocolate contained cocoa mass, cocoa butter, inulin, erythritol, natural vanilla extract, sunflower lecithin, and steviol glycosides, while conventional dark chocolate contained cocoa mass, sugar, cocoa butter, vanilla, and cocoa solids, as specified by manufacturers.

### Statistical analyses

Statistical analyses were performed in R (Version 3.6.2 “Dark and Stormy”) and GraphPad Prism (9.1.2). Baseline characteristics and questionnaire responses were summarized as mean (±SD) for continuous and N (%) for categorical data, unless otherwise stated. Normality was assessed by Shapiro-Wilk test. The pre-specified primary outcome was blood glucose incremental area under the curve (iAUC) including all timepoints from 0 (immediately before consumption of chocolate) to 120 min after consumption of chocolate. Secondary outcomes included peak blood glucose concentration (ie, highest blood glucose value measured after consumption of the chocolate), peak blood glucose concentration above baseline (ie, greatest positive difference between peak blood glucose value and fasting baseline glucose), and blood glucose total area under the curve (AUC). Effect of experimental condition was assessed using paired T-test (in the case of normally distributed data), or Wilcoxon’s Signed Rank test (in the case of non-normally distributed data).

## Results

### Participants

**Figure 1.**
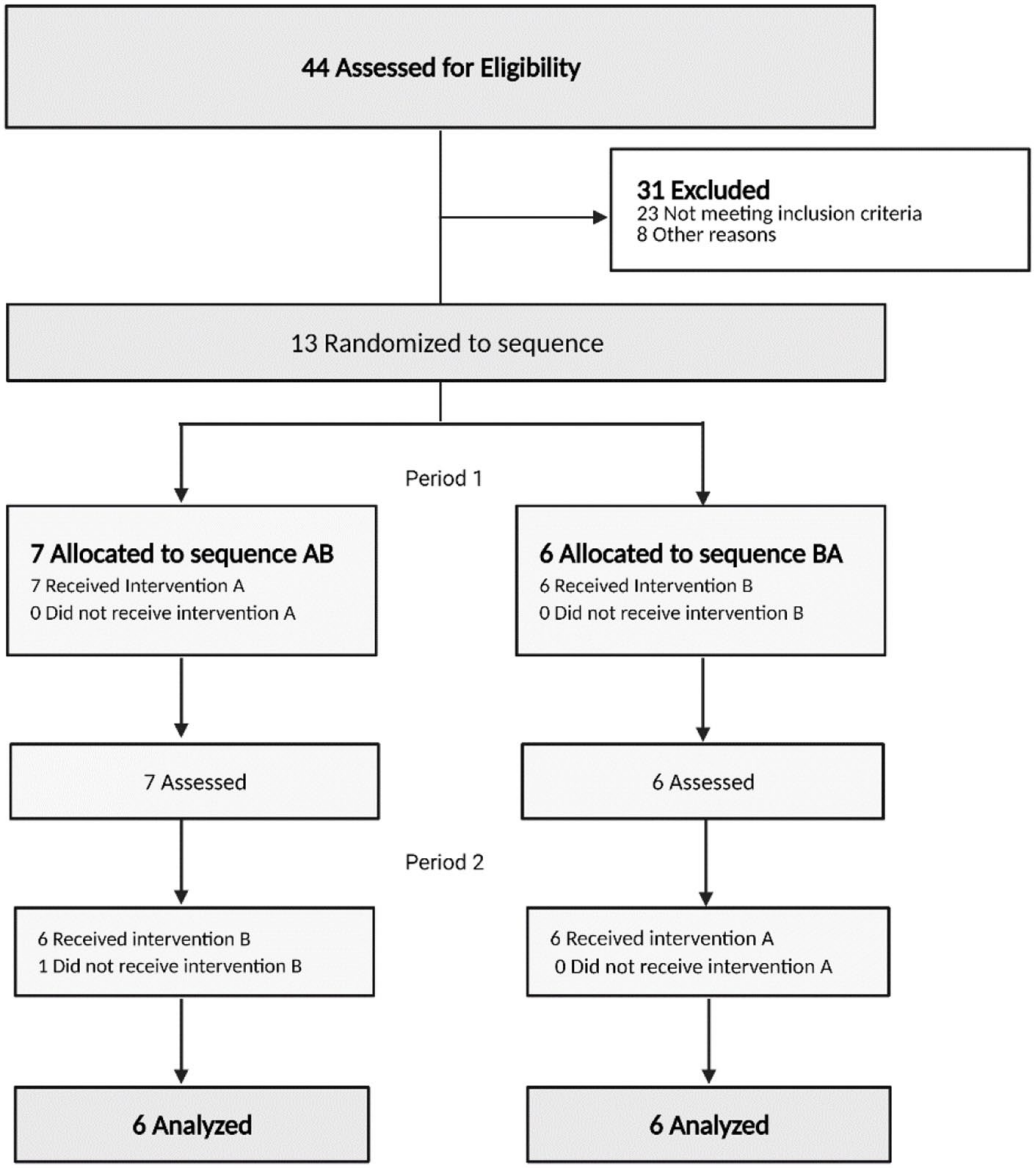
CONSORT flow. A = sugar-free dark chocolate; B = conventional dark chocolate.

From 13 participants (N = 6 T1D, N = 7 T2D), 12 completed both conditions. As shown in the Consort flow diagram in Figure 1, 44 participants were assessed for eligibility and 31 participants were excluded; 23 participants did not meet the inclusion criteria, and 8 participants were excluded for other reasons.

The participants (10 females and 3 males) were 51 ± 14 years old, had a body mass of 84.9 ± 23.3 kg, a body mass index (BMI) of 29.0 ± 5.6 kg/m^2^ and HbA1c of 7.3% ± 0.5%. Details on demographic and clinical characteristics are shown in Table 2.

**Table 2.** Self-reported baseline characteristics of participants.

Characteristic	Total	Type 1 diabetes	Type 2 diabetes
N	13	6	7
Female, n (%)	10 (77)	6 (100)	4 (57)
Age, mean (SD), years	51.1 (13.8)	47.5 (18.4)	54.1 (8.6)
Weight, mean (SD), kg	84.9 (23.3)	68.6 (6.1)	98.8 (23.7)
BMI, mean (SD)	29.0 (5.6)	25.3 (2.7)	32.2 (5.5)
HbA1c, mean (SD), %	7.3 (0.5)	7.2 (0.4)	7.4 (0.7)

### Glucose levels

**Figure 2.**
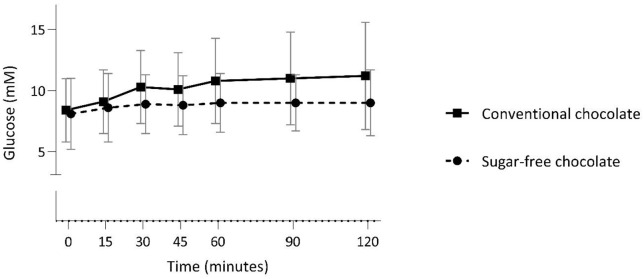
Glucose responses across 120 min after the consumption of a conventional dark chocolate bar and a sugar-free dark chocolate bar in participants with diabetes. Chocolate bars were consumed in the fasted state and blood glucose was assessed in finger prick samples. Values are means ± SD (N = 12).

Participants were asked to consume the chocolate in a fasted state based on the waythey were randomized, and to measure their blood glucose at baseline and after chocolate consumption at 15, 30, 45, 60, 90, and 120 min. The glucose response after consumption of the 2 chocolate bars is shown in Figure 2.

**Figure 3.**
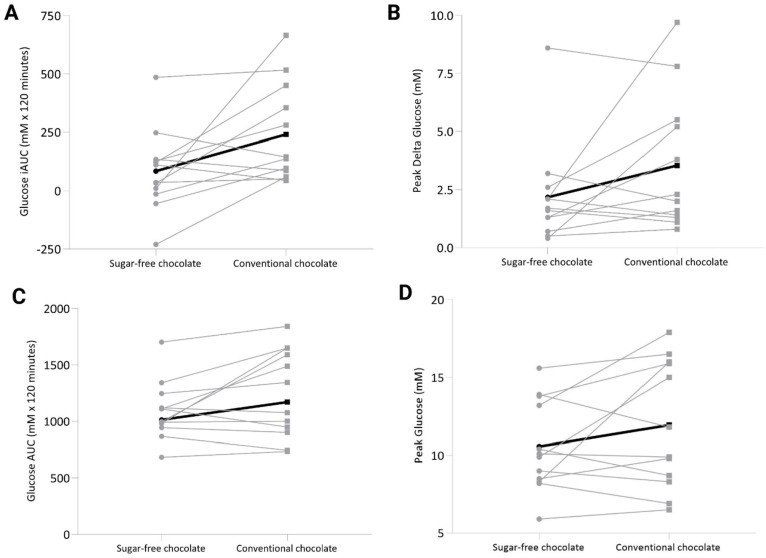
Glucose response to sugar-free versus conventional dark chocolate. Glucose response following consumption of either sugar-free (left) or conventional (right) dark chocolate is shown for (A) glucose incremental area under the curve, (B) peak glucose values above baseline, (C) glucose area under the curve, and (D) peak glucose values. Chocolates were consumed in a fasted state, and glucose was measured via finger prick after 15, 30, 45, 60, 90, and 120 min. Individual participant values are shown in the connected gray lines and the group mean is shown using the bolded black line. Conditions were compared using Wilcoxon Signed Rank Test (A and B) or Paired T-test (C and D). N = 12.

The primary outcome, incremental area under the curve (iAUC) was lower after consumption of sugar-free dark chocolate (−65%, *P* = .04) compared to conventional dark chocolate (Figure 3A). Although numerically lower in the sugar-free dark chocolate condition, no statistically significant differences were found for peak glucose value above baseline (−39%, *P* = .2; Figure 3B), total area under the curve (−13%, *P* = .07; Figure 3C), or peak glucose values (−11%, *P* = .15; Figure 3D).

## Discussion

This study demonstrates that consuming a sugar-free dark chocolate bar compared to a conventional dark chocolate bar has a lower impact on blood glucose excursions (iAUC) in participants with diabetes. To our knowledge, this is one of only a few studies that compares a sugar-free dark chocolate with a conventional dark chocolate on glucose outcomes in people with T1D and T2D.

Cacao, a component of dark chocolate rich in polyphenols, could be beneficial in preventing the onset of type 2 diabetes mellitus^12^ and improving vascular endothelial function.^13^ Frequent consumption of up to 2 to 6 servings (1 oz. or ~28 g each) of chocolate per week reduced the risk of diabetes and this effect appeared to show a dose-response relationship.^14^ Compared to white chocolate, polyphenol-rich dark chocolate decreased blood pressure and improved insulin sensitivity in healthy persons.^9^ Another study demonstrated that polyphenol-rich dark chocolate reduced fasting blood glucose levels in people with overweight and obesity.^15^ Many polyphenols found in chocolate, including epicatechin and catechin, have been found to alter glucose metabolism in cell culture and animal studies^16^ and it is hypothesized that polyphenols in dark chocolate have antioxidant effects that can improve endothelial function. However, not all studies demonstrate the benefits of dark chocolate on glucoregulatory outcomes, as dark chocolate containing 450 mg of polyphenols did not improve insulin and glucose responses in hypertensive participants with diabetes when administered 60 min prior to a 75 g oral glucose load.^6^ Rostami et al^17^ also found that daily consumption of 25 g of dark chocolate for 8 weeks did not improve fasting glucose, insulin, and HbA1c levels in hypertensive participants with diabetes. It stands to reason that the cardioprotective influence of cocoa ingestion could be attenuated by the sugar content of cocoa-containing products, particularly in people with diabetes.

The use of natural or artificial sweeteners offers a practical approach to support the reduction of sugar/carbohydrate intake for people with diabetes. By replacing sugars, people with diabetes may be able to maintain blood glucose control and help control body weight.^18^ The sugar-free dark chocolate used in this study contained a blend of stevia, erythritol, and inulin, in replacement for sugar. Stevioside has been associated with a reduction in postprandial blood glucose levels in people with T2D^19^ and stevia preloads significantly reduced postprandial glucose and insulin levels compared to sucrose preloads.^20^ Chukwuma et al^21^ have shown that erythritol significantly improved glucose tolerance in diabetic animals, especially 30 and 60 min after glucose ingestion. Erythritol also demonstrated antihyperglycemic effects in type 2 diabetic rats^22^ yet did not alter glucose homeostasis in people with glucose intolerance^23^ or diabetes^24^ in short-term studies. Erythritol is also classified as a sugar alcohol that is partially resistant to digestion and can also act as a dietary fiber. Although it is well absorbed from the small intestine compared to other sugar alcohols, it is not metabolized and thus, does not contribute to the systemic glucose pool.^25^ Therefore, in addition to mitigating the acute glycemic excursion after consumption, it is possible that the blend of sweeteners used in a sugar-free dark chocolate bar could have other potential health benefits for people with diabetes.

High-fiber diets have been established as a healthy option for the entire population and specifically for people with diabetes due, in part, to lower postprandial glucose levels.^26^ A recent meta-analysis supported the recommendation to increase dietary fiber intake in people with T2D in order to decrease HbA1c and fasting plasma glucose levels.^27^ Recommendations for adult dietary fiber intake generally fall in the range of 20 to 35 g/day.^28^ Fermentable fiber such as inulin may also have the potential to prevent and/or treat T1D.^29^ The sugar-free dark chocolate in this study was rich in fiber, mainly inulin, when compared to conventional dark chocolate (9 g in sugar-free and 5 g in conventional dark chocolate). Inulin is a prebiotic fiber and is low in calories. These multiple functional properties of inulin provide a potential advantage as an ingredient in chocolate formulations and could be an additional option to obtain the recommended daily fiber intake and support the ingestion of less calories.^30^

Chocolate is classified as a high-calorie food due to its high fat and sugar content. The sugar-free dark chocolate in this trial offered fewer calories compared to conventional dark chocolate due to the absence of sugar. Sugar substitutes have gained importance due to the increasing demand for diet products resulting from the growing effort to reduce the ingestion of added sugars. The link between body weight and T2D is strong, with studies confirming that the vast majority of people with T2D have overweight or obesity, and that people with obesity are at the highest risk of developing T2D. In addition, weight loss can prevent the development of T2D and improve glycemic control and other risk factors.^31^ Overweight and obesity have also increased among people with T1D and this elevates the risk of complications.^32-34^ Therefore, diets and food options lower in carbohydrates, sugars, and calories could be beneficial for people with T1D and T2D.

Our study is certainly not without limitations. First, due to the COVID-19 pandemic the study was conducted entirely virtual with no visits to the study site. Although this made it practical for participants and possible for us to collect data while in-person research was curtailed at our institution due to the COVID-19 pandemic, we cannot be entirely certain that participants followed the study protocol exactly and the data are not as reliable as if collected in a laboratory setting. To facilitate the recruitment of participants in a difficult research environment with limited time and budget, and to improve applicability, we included participants with both T1D and T2D, (despite the T1D group being only females) but were underpowered to make statistical comparisons between groups as this was not an aim of the study. Finally, this acute study involved a small sample size and consumption of only 1 bar of sugar-free or conventional dark chocolate so the longer-term effects on glucose control or other health parameters are unknown.

## Conclusions

A sugar-free (stevia, erythritol, and inulin-sweetened) dark chocolate bar led to a lower blood glucose iAUC compared to a conventional dark chocolate bar in people with diabetes. It appears that sugar-free dark chocolate could be consumed without compromising blood glucose control in people with diabetes. Longer-term trials with greater sample sizes will be needed to assess if consumption of sugar-free dark chocolate could have cardiovascular health benefits while improving or not worsening glucose control in diabetes.
